# Racial and ethnic disparities in medication adherence among privately insured patients in the United States

**DOI:** 10.1371/journal.pone.0212117

**Published:** 2019-02-14

**Authors:** Zhiwen Xie, Patricia St. Clair, Dana P. Goldman, Geoffrey Joyce

**Affiliations:** 1 Schaeffer Center for Health Policy & Economics, University of Southern California, Los Angeles, California, United States of America; 2 Data Core, Schaeffer Center for Health Policy & Economics, University of Southern California, Los Angeles, California, United States of America; 3 Schools of Public Policy, Pharmacy, and Economics, University of Southern California, Los Angeles, California, United States of America; 4 Department of Pharmaceutical & Health Economics, USC School of Pharmacy, Los Angeles, California, United States of America; University of Arizona, UNITED STATES

## Abstract

**Objective:**

To examine the association between socioeconomic status (SES) and racial and ethnic disparities in medication adherence for three widely prescribed therapeutic classes

**Methods:**

We linked longitudinal claims data from a large US-based insurance provider (2011–2013) to detailed SES information to identify patients treated with oral antidiabetic (N = 56,720), antihypertensive (N = 156,468) or antihyperlipidemic (N = 144,673) medications. We measured adherence and discontinuation by therapeutic class, and conducted regression analysis to quantify the contributions of different factors in the association between race/ethnicity and medication adherence.

**Results:**

During an average follow-up period of 2.5 years, average adherence rates of Blacks and Hispanics were at least 7.5 percentage points lower than those of Whites. Controlling for demographics, health status, out-of-pocket costs, convenience of refilling prescriptions and SES attenuated the association by 30 to 50 percent, nonetheless substantial racial disparities persisted (4.1–5.8 percentage points), particularly for asymptomatic conditions. Separating adherence among existing users from those that discontinued therapies indicates that racial/ethnic disparities in adherence reflect inconsistent pill-taking rather than differential rates of discontinuation.

**Conclusions:**

Racial/ethnic disparities in adherence are mitigated, but persist after controlling for detailed socioeconomic measures. Interventions should focus more on improving medication adherence of existing users, particularly in treating asymptomatic conditions.

## Introduction

Chronic medications must be taken as prescribed to be effective, yet poor adherence is endemic [1–10]. Nearly half of all patients that were prescribed pharmaceutical therapies do not take sufficient doses to experience therapeutic benefits [11]. Suboptimal medication-taking behavior, consisting of poor adherence among existing users and discontinuation of therapy, is particularly acute in racial/ethnic minority populations. Minorities typically have higher prevalence of chronic disease, worse access to medical care, and greater financial constraints [12–15]. Past studies suggest that Blacks and Hispanics are at least 50 percent more likely to have suboptimal adherence rates than Whites, and that differences in socioeconomic status (SES) may play a significant role in explaining these deficits [5,16].

A large number of U.S. studies have documented suboptimal medication adherence and discontinuation across a wide range of patient populations and disease conditions, but few have examined the association after controlling for detailed measures of socioeconomic status such as income and education [9,10,13,16]. Studies that rely on pharmacy claims usually lack information on a patient’s race/ethnicity and socioeconomic well-being, at best inferring differences in SES from averages of large geographic units such as zip codes or census tracts [6,7,16]. By contrast, studies that rely on survey data include more accurate and detailed sociodemographic information, but rely on self-reported adherence measures, which are shown to overestimate actual adherence [12,17–19].

In this paper, we linked longitudinal pharmacy and medical claims to detailed demographic and socioeconomic data on privately insured patients to compare race-specific rates of medication adherence and discontinuation to commonly prescribed medications used to treat hypertension, hyperlipidemia, and Type 2 diabetes mellitus (T2DM). We also seek to identify the impacts of adjusting for socioeconomic status (SES) on the association between race/ethnicity and medication adherence, which can inform appropriate interventions to help reduce disparities in adherence and health outcomes.

## Methods

### Data & sample selection

This study used a 25% sample of De-identified Clinformatics Data Mart (OptumInsight, Eden Prairie, MN), a large-scale claims dataset of privately insured patients with both medical and pharmacy coverage from a large commercial insurer in the United States from 2011 to 2013.

We linked enrollment data to medical and pharmacy claims over time for commercially insured members and their dependents. The enrollment data included information on patients’ plan type, coverage period, gender, and year of birth. The prescription claims included information on the date of each prescription, type of drug, brand and generic name, active ingredient, and days of supply, as well as financial information such as the copayment and deductible associated with each claim. The medical claims captured standard information, including date of service, diagnosis codes, and duration of hospital stays.

Our study focused on chronic medications treating T2DM, hypertension, and hyperlipidemia, three of the most prevalent and costly chronic conditions in the United States [20]. We identified patients taking medications for these conditions by examining their prescription drug claims and enrollment files. We selected individuals age 18 to 64, not enrolled in a Medicare Advantage plan. To ensure at least a year of follow-up, we included individuals who filled two or more prescriptions in the therapeutic class in 2011 or 2012 and were continuously enrolled in the same benefit plans through December 31^st^, 2013. Based on these inclusion criteria, our study sample consisted of 56,720 (3.9 percent) beneficiaries taking oral antidiabetics, 156,468 (10.9 percent) beneficiaries taking antihypertensive medications, and 144,673 (10.1 percent) beneficiaries taking antihyperlipidemic medications (See Table A1 in S1 File for details of sample selection). The average follow-up period was 2.4, 2.5, and 2.5 years for patients on oral antidiabetic, antihypertensive, and antihyperlipidemic medications, respectively.

### Socioeconomic variables & race/ethnicity

In addition to the standard demographic information, the claims data were linked to a detailed set of SES measures for each beneficiary, including race/ethnicity, education level, and household income. Race was either self-reported or derived from a combination of public records, purchase transactions, and consumer surveys [21]. Education at the census block group level was derived from U.S. Census data. (Census block group is the smallest unit on which the Census bureau publishes sample data. It has a population of 600 to 3,000 people. It is smaller than a Census Track, and larger than a Census Block.) OPTUMInsight has ensured that the dataset is statistically de-identified and consistent with all HIPAA requirements.

### Adherence measures–proportion of days covered (PDC) and discontinuation

Poor adherence can be a product of three different behavioral pathways: reduced initiation of treatment, worse adherence among existing users, and/or discontinuation of therapy. Since we could not infer primary nonadherence (patients who were prescribed a medication but never initiated therapy), we focused on secondary adherence of patients after they initiated a therapy [22–24]. We used proportion of days covered (PDC) to measure adherence behavior of current users. PDC is a well-established method for measuring adherence using claims data [22,23,25,26] and offers a more conservative estimate compared to the medication possession ratio (MPR) [22,27]. We measured adherence at the effective ingredient level. In cases where a patient was on multiple medications within the same therapeutic class, the patient was considered adherent for a particular day if he or she was covered by at least one of the medications within the therapeutic class. We defined the observation period from the date of first fill within the therapeutic class to the last day of 2013, excluding days when the patient was hospitalized.

To more accurately reflect adherence among active users, we identified patients who discontinued therapy altogether. Patients were considered to have discontinued a therapy if they were not in possession of any drug in the class for at least 180 days. By this definition, 24 to 26 percent of patients discontinued treatment in the approximately 30-month follow-up period (S1 Table). For patients that discontinued a therapy, we measured their adherence only for the period when they were taking the medication, defined as the period from the date of the first observed fill to the last day of possession in the follow-up period.

### Statistical analysis–multivariate regressions

We first examined differences in adherence by race/ethnicity in each therapeutic class. To understand the contribution of different observable factors in racial/ethnic disparities in adherence rates [28], we performed stepwise linear regressions. We started with a baseline specification with patient-level PDC as the dependent variable and binary indicators for each race/ethnicity. In turn, we added: patient demographics (age in log form, gender and geographic area of residence defined by census division), comorbid conditions (Charlson comorbidity index, and other chronic drugs taken within the observation period (on an active ingredient level)), out-of-pocket (OOP) costs (calculated as the average copay and deductible per day of supply of medication), percentage of medication obtained through mail order pharmacy, average days of supply per prescription, education (categorical variable), and household income (categorical variable). Education and income were entered last, as one of our primary objectives was to examine how SES variables moderated the association between race/ethnicity and medication adherence after controlling for patient demographics and insurance status. Throughout this process, we observed how racial/ethnic differences in adherence rates changed with additional controls and whether these changes varied across the three therapeutic classes. We hypothesized that controlling for a richer set of socioeconomic variables would further weaken the racial and ethnic disparities in medication adherence. While this exercise cannot establish a causal relationship, it provides an estimate of the relative effects of SES compared to standard demographic controls, comorbidities and plan characteristics, including patient cost-sharing and convenience of refilling medications.

As a second step, we performed two sets of logistic regression models, by therapeutic class, to quantify and compare the relative risk of suboptimal adherence (PDC < 0.8) and the likelihood of discontinuing therapy altogether. These models adjusted for all the covariates included in the previous analysis. The regression coefficients were then used to predict odds ratios by race/ethnicity of suboptimal adherence and discontinuation.

All statistical analyses were conducted using Stata version 14.1.

## Results

S1 Table shows the characteristics of the study sample, by therapeutic class. Overall, 56 percent of patients in the sample were male, with an average age of 52.6 years. While the majority were white, Blacks and Hispanics made up a disproportionate share of those taking oral antidiabetic medications. Users of oral antidiabetic medications had lower levels of educational attainment and household income, and more comorbidities than those taking antihypertensive or cholesterol-lowering medications. Nearly three-fourths of the study sample were enrolled in a point of service plan.

S2 Table shows average (unadjusted) adherence rates by race, education and income in each therapeutic class. Average adherence was highest among patients taking antihypertensives (78.4 percent, oral antidiabetic 74.7 percent, antihypertensive 75.3 percent), and among those taking medications in 2 or 3 of the classes analyzed (35.7%). Further, there were significant differences by race/ethnicity, education and income. Non-White, low educational attainment, and low household income were associated with lower adherence rates in all three classes. For example, the average adherence rate among whites taking an oral antidiabetic was 8.4 percentage points higher than Hispanics, which is equivalent to one less month (31 days) of medication use per year. The mean PDC of patients in the lowest income bracket (<$40K) was 9.4 percentage points lower than those in the highest income bracket (>$100K), equivalent to 34 less days covered by medication in a year.

S3 Table shows how racial/ethnic gradients in adherence changed as we added additional sets of covariates. The baseline model reports unadjusted differences for each racial/ethnic group relative to whites, similar to S2 Table. For example, the average PDC of Hispanics is 7.9 to 9.2 percentage points lower than whites. Similarly, the average PDC of blacks is 7.5 to 8.7 percentage points lower than whites without adjustment. Controlling for the full set of covariates reduces these differences by 30% to 50% across the three drug classes. For example, the average PDC of Hispanics is 4.1 to 5.8 percentage points lower than whites after adjustment, while the black-white differential is reduced to 4.3 to 5.2 percentage points. Average adherence rates of Asians are only modestly lower than whites before adjustment, and remain similar in absolute terms in the full model. The average PDC of Asians is 1.1 to 3.3 percentage points lower than whites after adjustment.

S4 Table compares the adjusted odds of discontinuing therapy and having suboptimal adherence (PDC <0.8) for each race/ethnicity relative to whites, by therapeutic class. Blacks and Hispanics are more likely to discontinue an antihyperlipidemic (odds ratio of 1.15, 1.30 respectively), but not antihypertensive or antidiabetic medications after adjustment. On the other hand, blacks and Hispanics actively using these chronic medications are 36 to 59 percent more likely to have poor adherence than Whites, defined as a PDC < .80.

## Discussion

We found that controlling for socioeconomic characteristics reduced, but did not eliminate the association between race/ethnicity and medication adherence. Average adherence rates of blacks and Hispanics were 4.8 to 6.5 percentage points lower than whites across three widely used drug classes, which translates to about 20–25 fewer days of medication use per year. We found smaller racial/ethnic differences in adherence to medications for more symptomatic conditions such as antidiabetics, wherein the consequences of poor adherence are more evident to the patient. While blacks and Hispanics take these medications less consistently than whites, they were not at higher risk for discontinuing a medication, although the results varied by therapeutic class. This suggests that reducing disparities in adherence should focus on improving daily adherence rather than cessation of therapy.

Prior work has shown that higher adherence is associated with better management of chronic conditions (A1C, LDL, and blood pressure), lower hospitalizations, and reduced mortality risk [29–33]. For example, Ho et al found that nonadherence to antihypertensive and antihyperlipidemic medications was associated with a 10 to 40 percent relative increase in risk of cardiovascular hospitalizations and a 50 to 80 percent higher risk of mortality [33]. Thus, even modest improvements in the medication adherence of minorities has potential to narrow observed differences in health across race/ethnicity.

Our findings are generally consistent with the previous literature, but smaller in magnitude [5,6,16]. This is likely due to better controls for socioeconomic status, which has been shown to be strongly correlated with better health and health behaviors [34,35]. We also hypothesized that racial/ethnic differences in adherences may be driven by differential rates of discontinuation. Patients commonly discontinue therapy, either at the direction of their provider, or more commonly, on their own due to side-effects, perceived ineffectiveness, distrust of physician’s recommendations, or cost [9,36–38]. Prior work typically measures adherence over a pre-determined period of time, such as a calendar year or twelve months after an initial fill date, underestimating overall adherence rates and often categorizing those who have stopped taking a medication as poor adherers. While this is an important methodological distinction, it did not explain differences in average adherence by race/ethnicity. Previous studies have also shown that the convenience and duration of getting prescription refills was associated with improved adherence [16,36,39]. In our study, we also found that black and Hispanic patients had fewer 90-day fills and less frequent use of mail-order pharmacies than whites and Asians (Tables A6 and A7 in S1 File). Controlling for average days of supply per fill and the use of mail-order pharmacy further attenuated racial/ethnicity disparities, but significant differences persisted.

Our study has several limitations. First, while pharmacy claims have been widely used to estimate adherence, they do not measure actual pill-taking behavior. Second, our study sample consists of younger, privately insured patients of above-average SES. Thus, our results may not generalize to lower SES groups such as a Medicaid population. Third, we measure adherence among those actively using a medication, excluding periods when patients have discontinued therapy for at least 6 months. We conservatively used 180 days, but there is no standard definition of discontinuation or persistence to a chronic medication. Using this definition, we found that about 1 in 5 patients discontinued their chronic medication over an average follow-up of 2.5 years. Defining discontinuation based on a gap of 90 days or more reduced the number of patients stopping therapy and lowered average adherence rates, but did not substantively change adherence rates differentially across race/ethnicity. Lastly, our regression results do not yield a causal explanation for non-adherence, rather they provide estimates of the effects of education, income and race separately on adherence, which can guide potential interventions for moderating these differences.

## Public health implications

Our findings indicate that non-white patients were no more likely to discontinue a medication than whites; rather, they took their chronic medications less consistently. While changing patient behavior is difficult, prescription drug claims and electronic medical records allow for real-time interventions via text message or low-cost reminder devices that could prove impactful for non-White patients who have gaps in coverage. Alternatively, such data could be monitored to identify patients who did not refill a medication in a timely fashion, eliciting a call from a pharmacist, nurse or health care provider to understand the reason for nonadherence and intervene if appropriate. The combination of real-time pharmacy claims and the near universal adoption of electronic devices could potentially prove more cost-effective than previous approaches to improving adherence, although there is no conclusive evidence on the long-term effectiveness of patient reminders [40,41].

## Supporting information

S1 Table Characteristics of patients by therapeutic class. (DOCX)

S2 Table Average adherence rates (PDC) by race, education and household income. (DOCX)

S3 Table Contribution of factors towards explaining the racial gradient in medication adherence. (DOCX)

S4 Table Adjusted odds ratio of discontinuation and nonadherence (PDC<0.8) by race/ethnicity. (DOCX)

S1 File Supplemental tables. (XLSX)

## Abbreviations

SES: Socioeconomic Status
PDC: Proportion of Days Covered
MPR: Medication Possession Ratio
T2DM: Type 2 Diabetes Mellitus
OOP: Out-of-Pocket
