# Supplementary material for: Racial and ethnic disparities in medication adherence among privately insured patients in the United States
Source: PLoS One. 2019 Feb 14;14(2):e0212117. doi: 10.1371/journal.pone.0212117 (PMC6375669; doi:10.1371/journal.pone.0212117)
Supplement: S1 Table — (DOCX) [file pone.0212117.s001.docx]

| **Table 1 – Characteristics of Patients by Therapeutic Class** | | | |
| --- | --- | --- | --- |
| Therapeutic Class | Oral Antidiabetic  (N=56,720) | Antihypertensive  (N=156,468) | Antihyperlipidemic  (N=144,673) |
| Follow-up Period^a^, mean, y | 2.4 | 2.5 | 2.5 |
| Discontinued, % | 24 | 26 | 24 |
| Age, mean (SD), y | 52.0 (9.4) | 52.9 (8.5) | 54.2 (7.5) |
| Male, % | 51 | 57 | 60 |
| **Race, %** |  |  |  |
| White | 67 | 74 | 79 |
| Asian | 4 | 3 | 3 |
| Black | 15 | 14 | 10 |
| Hispanic | 13 | 9 | 8 |
| **Education, %** |  |  |  |
| College Degree | 12 | 15 | 18 |
| Some College | 52 | 53 | 54 |
| High School and Less | 36 | 32 | 28 |
| **Annual Household Income, %** |  |  |  |
| >$100K | 28 | 44 | 49 |
| $75K-$99K | 18 | 18 | 18 |
| $60K-$74K | 13 | 12 | 11 |
| $50K-$59K | 9 | 8 | 7 |
| $40K-$49K | 8 | 7 | 6 |
| <$40K | 14 | 11 | 9 |
| **Insurance Plan Type, %** |  |  |  |
| Point of Service (POS) | 74 | 74 | 75 |
| Exclusive Provider Organization (EPO) | 15 | 14 | 13 |
| Health Maintenance Organization (HMO) | 9 | 9 | 9 |
| Others (PPO, Indemnity and others) | 2 | 3 | 3 |
| Charlson Comorbidity Index, mean (SD) | 2.3 (2.0) | 1.4 (1.8) | 1.3 (1.8) |

Note. Percentages of patients in each group of race/ethnicity and SES variables (education and annual household income) are calculated based on patients with known race/ethnicity and SES information. The percentages of patients with unknown race/ethnicity and SES information are reported in supplement table A1.

^a^Follow-up period for a therapeutic class is defined as the period from the first fill (in either 2011 or 2013) until Dec. 31^st^, 2013.
