# Supplementary material for: Racial and ethnic disparities in medication adherence among privately insured patients in the United States
Source: PLoS One. 2019 Feb 14;14(2):e0212117. doi: 10.1371/journal.pone.0212117 (PMC6375669; doi:10.1371/journal.pone.0212117)
Supplement: S2 Table — (DOCX) [file pone.0212117.s002.docx]

| **Table 2 – Average Adherence Rates (PDC) by Race, Education and Household Income** | | | |
| --- | --- | --- | --- |
| **Therapeutic Class** | **Oral Antidiabetic**  (N=56,720) | **Antihypertensive**  (N=156,468) | **Antihyperlipidemic**  (N=144,673) |
| All, mean (SD) | 74.7 (24.3) | 78.4 (22.2) | 75.3 (22.6) |
| ***By Race*** |  |  |  |
| White | 76.9 | 80.2 | 77.0 |
| Asian | 74.3 | 77.5 | 72.9 |
| Black | 69.4 | 72.5 | 68.4 |
| Hispanic | 68.5 | 72.3 | 67.9 |
| ***By Education*** |  |  |  |
| College Degree | 78.6 | 81.9 | 78.4 |
| Some College | 75.4 | 79.1 | 75.9 |
| High School and Less | 72.2 | 75.8 | 72.1 |
| ***By Household Income*** |  |  |  |
| HH Income > $100K | 78.4 | 81.4 | 77.7 |
| $75K-$99K | 75.7 | 79.0 | 75.5 |
| $60K-$74K | 74.2 | 77.6 | 74.2 |
| $50K-$59K | 73.1 | 76.2 | 72.9 |
| $40K-$49K | 72.1 | 75.3 | 71.7 |
| <$40K | 69.0 | 73.2 | 69.4 |
