# Supplementary material for: Racial and ethnic disparities in medication adherence among privately insured patients in the United States
Source: PLoS One. 2019 Feb 14;14(2):e0212117. doi: 10.1371/journal.pone.0212117 (PMC6375669; doi:10.1371/journal.pone.0212117)
Supplement: S3 Table — (DOCX) [file pone.0212117.s003.docx]

| **Table 3. Contribution of Factors Towards Explaining the Racial Gradient in Medication Adherence** | | | |
| --- | --- | --- | --- |
|  | **Oral Antidiabetic** | **Antihypertensive** | **Antihyperlipidemic** |
|  | **Average PDC for White Patients, %** | | |
|  | 76.9 | 80.2 | 77.0 |
| **Covariates in the Model** | **Differences in Average PDC between Hispanics and Whites** ^b^**, %** | | |
| Baseline ^a^ | -8.4 | -7.9 | -9.2 |
| (1) Demographics (Gender, Age, Geographic Area) | -6.6 | -6.6 | -7.8 |
| (2) = (1) + Comorbidities (Charlson Index, Other Drugs Taken) | -6.0 | -6.2 | -7.5 |
| (3) = (2) + Out-of-Pocket (OOP) Cost | -6.0 | -6.2 | -7.6 |
| (4) = (3) + Mail Order^c^ + Average Days of Supply Per Refill | -4.9 | -5.3 | -6.6 |
| (5) = (4) + Education | -4.4 | -4.9 | -6.1 |
| (6) = (5) + Income | -4.1 | -4.6 | -5.8 |
|  | **Differences in Average PDC between Blacks and Whites** ^b^**, %** | | |
| Baseline ^a^ | -7.5 | -7.8 | -8.7 |
| (1) Demographics (Gender, Age, Geographic Area) | -6.4 | -6.6 | -7.2 |
| (2) = (1) + Comorbidities (Charlson Index, Other Drugs Taken) | -6.0 | -6.3 | -7.1 |
| (3) = (2) + OOP Cost | -6.0 | -6.2 | -7.2 |
| (4) = (3) + Mail Order^c^ + Average Days of Supply Per Refill | -5.1 | -5.4 | -6.2 |
| (5) = (4) + Education | -4.7 | -5.0 | -5.7 |
| (6) = (5) + Income | -4.3 | -4.5 | -5.2 |
|  | **Differences in Average PDC between Asians and Whites** ^b^**, %** | | |
| Baseline ^a^ | -2.6 | -2.8 | -4.2 |
| (1) Demographics (Gender, Age, Geographic Area) | -0.7 ^d^ | -2.1 | -3.1 |
| (2) = (1) + Comorbidities (Charlson Index, Other Drugs Taken) | 0.1 ^d^ | -1.7 | -2.7 |
| (3) = (2) + OOP Cost | 0.1 ^d^ | -1.7 | -2.8 |
| (4) = (3) + Mail Order^c^ + Average Days of Supply Per Refill | -0.1^d^ | -2.1 | -2.9 |
| (5) = (4) + Education | -1.2 | -2.5 | -3.4 |
| (6) = (5) + Income | -1.1 | -2.5 | -3.3 |

Note. This table shows how racial differences in adherence evolved as we added in more controls into the model. In each step, the controls included all the controls from previous steps.

^a^ Baseline gap in PDC are the difference in average (unadjusted) in PDC between racial groups. The averages for different racial groups were reported in Table 2.

^b^ Differences in average PDC are calculated as the differences relative to Whites. A negative value indicates the group has lower average PDC than Whites.

^c^ Mail order is measured by the percentage of days of supply obtained through mail order among all days of supply used to calculate PDC at a patient level.

^d^ p-value>0.1 for all these differences, and p-value<0.01 for all others.
