# Supplementary material for: Racial and ethnic disparities in medication adherence among privately insured patients in the United States
Source: PLoS One. 2019 Feb 14;14(2):e0212117. doi: 10.1371/journal.pone.0212117 (PMC6375669; doi:10.1371/journal.pone.0212117)
Supplement: S4 Table — (DOCX) [file pone.0212117.s004.docx]

| **Table 4. Adjusted Odds Ratio of Discontinuation and Nonadherence (PDC <0.8) by Race/Ethnicity** | | | | | |
| --- | --- | --- | --- | --- | --- |
|  | **% of Patients**  **That Discontinued** | **Adjusted Odds Ratio**  **Of Discontinuation** |  | **% of Nonadherent**  **Patients (PDC < 0.8)** | **Adjusted Odds Ratio**  **Of Being Nonadherent** |
|  | **Oral Antidiabetic** | | | | |
| White | 23.0 | 1 (Reference) |  | 43.1 | 1 (Reference) |
| Asian | 22.1 | 0.80 (0.72-0.90) |  | 48.2 | 1.13 (1.03-1.24) |
| Black | 24.8 | 0.90 (0.85-0.96) |  | 56.7 | 1.37 (1.30-1.45) |
| Hispanic | 26.5 | 0.98 (0.92-1.04) |  | 58.2 | 1.36 (1.29-1.44) |
|  | **Antihypertensive** | | | | |
| White | 24.6 | 1 (Reference) |  | 36.1 | 1 (Reference) |
| Asian | 24.4 | 1.00 (0.93-1.08) |  | 41.2 | 1.22 (1.14-1.31) |
| Black | 29.0 | 1.02 (0.98-1.06) |  | 51.8 | 1.47 (1.42-1.52) |
| Hispanic | 28.9 | 1.06 (1.02-1.11) |  | 51.3 | 1.44 (1.38-1.49) |
|  | **Antihyperlipidemic** | | | | |
| White | 22.1 | 1 (Reference) |  | 43.1 | 1 (Reference) |
| Asian | 25.2 | 1.13 (1.06-1.22) |  | 51.9 | 1.35 (1.26-1.43) |
| Black | 29.2 | 1.15 (1.10-1.20) |  | 58.4 | 1.45 (1.39-1.50) |
| Hispanic | 31.5 | 1.30 (1.25-1.36) |  | 60.4 | 1.59 (1.52-1.65) |

Note. Patients are considered to have discontinued the medications if they are not uncovered by any medication in the last 180 days of the follow-up period. Adjusted odds ratios of discontinuation and being nonadherence are both calculated based on our multivariate logistic regression models, adjusting for demographics, comorbidites, out-of-pocket cost, average days of supply per refill, percentage of days of supply refilled through mail order, and SES.
